# Design, Development, and Testing of an Intelligent Wearable Robotic Exoskeleton Prototype for Upper Limb Rehabilitation

**DOI:** 10.3390/s21165411

**Published:** 2021-08-10

**Authors:** Manuel Andrés Vélez-Guerrero, Mauro Callejas-Cuervo, Stefano Mazzoleni

**Affiliations:** 1 Software Research Group, Universidad Pedagógica y Tecnológica de Colombia, Tunja 150002, Colombia; mauro.callejas@uptc.edu.co; 2 School of Computer Science, Universidad Pedagógica y Tecnológica de Colombia, Tunja 150002, Colombia; 3 Department of Electrical and Information Engineering, Polytechnic University of Bari, 70126 Bari, Italy; stefano.mazzoleni@poliba.it

**Keywords:** robotic exoskeletons, wearable devices, artificial intelligence (AI), artificial neural networks (ANN), adaptive algorithms, upper limbs, rehabilitation, healthcare, control strategies

## Abstract

Neuromotor rehabilitation and recovery of upper limb functions are essential to improve the life quality of patients who have suffered injuries or have pathological sequels, where it is desirable to enhance the development of activities of daily living (ADLs). Modern approaches such as robotic-assisted rehabilitation provide decisive factors for effective motor recovery, such as objective assessment of the progress of the patient and the potential for the implementation of personalized training plans. This paper focuses on the design, development, and preliminary testing of a wearable robotic exoskeleton prototype with autonomous Artificial Intelligence-based control, processing, and safety algorithms that are fully embedded in the device. The proposed exoskeleton is a 1-DoF system that allows flexion-extension at the elbow joint, where the chosen materials render it compact. Different operation modes are supported by a hierarchical control strategy, allowing operation in autonomous mode, remote control mode, or in a leader-follower mode. Laboratory tests validate the proper operation of the integrated technologies, highlighting a low latency and reasonable accuracy. The experimental result shows that the device can be suitable for use in providing support for diagnostic and rehabilitation processes of neuromotor functions, although optimizations and rigorous clinical validation are required beforehand.

## 1. Introduction

Even with advances in health sciences, neuromotor dysfunction resulting in human limb limitations is still prevalent worldwide [1]. In 2011, the population living with disabilities due to neuromotor dysfunction was around 15% [2]. It is estimated that by 2021, more than two billion people will be living with some form of disability, equivalent to approximately 37.5% of the global population [3,4]. It is clear that numbers have increased and will continue rising over the years, representing a significant proportion of the population in all age groups.

### 1.1. Understanding Disabilities and the Rehabilitation Process

The population in a disability condition due to musculoskeletal or neuromotor dysfunction has been disproportionately affected due to the COVID-19 pandemic, its demographic trends, and the increase in associated chronic diseases [5]. For this reason, it is necessary to extend services for people with disabilities, establish rehabilitation interventions as a priority [6], and provide concerted and sustained efforts to enhance the treatments [7,8]. The Disability and Development Report [9] and the Report on the disability inclusion in the United Nations system [10] note that the deployment of assistive technologies must be accompanied by planning based on quantitative data to obtain valuable information synchronized with the rehabilitation requirements [11,12].

The incidence of stroke injuries remains among the main causes that can lead to disability or neuromotor dysfunction [13] therefore, covering the evolution of physical and neuromotor rehabilitation treatments can lead to solutions for patients affected by other causes [14]. The injuries created by any pathology disrupt the functional performance of the patient, creating social and daily life implications [15], hence recovery focused on the development of activities of daily living (ADLs) has been the basis for the creation of different and better rehabilitation schemes, highlighting traditional physical therapy sessions and technology-assisted processes [16]. 

There are some drawbacks inherent in the traditional approach to rehabilitation, where the procedures often lack comprehensive coverage due to the partially subjective and limited experience of physicians [17,18], and the time lag between the injury event and the initiation of treatment [19]. In many cases, the injury is not addressed in terms of neuromotor functioning, limiting the patient’s progress [20]. For this reason, it is suggested to treat each patient individually [21,22], but when the optimal actions to be taken are not clear and the psychological and emotional aspects of the therapies are not considered [23], a slow, costly and complex rehabilitation process occurs [24].

Recent advances in assistive rehabilitation technologies have identified continuous training using robots and objective specialist assessments as an effective way to rebuild lost neuromotor plasticity [25,26]. However, there are currently some barriers that hinder access to existing developments, such as low coverage and affordability of the devices and the lack of financial resources [9]. The availability of assistive products is also limited in terms of quantity, models, and sizes, thus further research on these features is still needed [27]. Despite these drawbacks, rehabilitation support technologies are becoming increasingly accessible, moving the rehabilitation environment from specialized centers into patients’ everyday spaces [28].

### 1.2. Proposal and Organisation

Taking into account the growing need to design systems that contribute to the solution of existing problems in rehabilitation using emerging technologies, it is proposed as a research objective the development of an autonomous and wearable robotic exoskeleton prototype using an active orthosis, different data acquisition systems, and a state-of-the-art Artificial Intelligence (AI) core. Moreover, mixing the advantages of the leading technological approach and the benefits of specialist-based training sessions, this proposed prototype contributes to comprehensive neuromotor assessment and assistance in the arm and forearm segment rehabilitation, trying to minimize the factors that hinder the recovery process in the patient.

In the following sections, this paper will discuss the design, development, and preliminary validation of the described robotic exoskeleton prototype with fully embedded and autonomous AI-based control, processing, and safety algorithms. The prototype has been designed using semi-flexible, ergonomic, and low-cost materials, resulting in a lightweight, portable and versatile exoskeleton. This exoskeleton is proposed as a compact system with one Degree-of-Freedom (1-DoF) in the elbow joint, allowing both flexion and extension movements. The implementation of 1-DoF allows for a functional proof of concept focusing on the ergonomic and practical design for rehabilitation processes without neglecting the clinical importance, developing a suitable device for patients with different pathologies that cause partial or complete disability of the upper limbs [29,30].

The sections of this paper are organized as follows: Section 2 introduces a brief review of the state-of-the-art focusing on a compact, wearable, and intelligent exoskeletons for upper limbs. Section 3 presents the materials and methods to design and develop the exoskeleton, as well as the control strategies and operation modes. Section 4 describes the main results of functional testing in a laboratory environment. 

Finally, the paper ends with a further result discussion in Section 5, while the conclusion and future work is described in Section 6.

## 2. Related Works

Previous studies have addressed upper limb rehabilitation assistance processes using advanced technology, leading to the development of sophisticated robotic exoskeletons [31,32]. These developments integrate a growing number of sensors and techniques that allow the proper application of training and physical conditioning sessions [33], also establishing a prominent referential source that influences the present work. The most relevant developments in the area are described below.

### 2.1. Rehabilitation Exoskeleton Devices

In general, some reviews have shown recent advances developed all over the world in rehabilitation technology applied to different segments of the human body. The paper presented by De La Tejera, et al. [34] stands out as a good starting point to understand the different classifications and types of robotic exoskeletons available today. This article provides an overview of their use in rehabilitation along with an analysis of the control techniques used in the devices.

A more exhaustive review is provided in [35], which focuses on upper limb devices, stating the more challenging aspects of developing assistive exoskeletons at the mechatronics and control level. Finally, other reviews specialize in particular topics [36,37], gathering developments involving new control techniques based on intelligent algorithms and also new mechanical and physical development techniques through the use of pneumatic actuators, electrical actuators, and the use of new materials and manufacturing processes.

### 2.2. Intelligent Exoskeleton Devices

Rehabilitation robotic exoskeletons that include AI-based control or information fusion techniques are proposed as the evolution of traditional exoskeletons. Noteworthy are implementations using Artificial Neural Networks (ANNs) [38,39] or adaptive algorithms [40,41], allowing the early pattern recognition that leads to an enhanced assessment of patients and their behavior with the devices [42,43]. The most remarkable developments in this field [44,45] are using machine learning to detect motion intentions during rehabilitation, making it possible to adjust the physical therapy session according to some of the particular needs of each patient.

Further developments using intelligent algorithms improve the mechanical performance of exoskeletons [46,47]. High-performance, disturbance-tolerant systems stand out for their reliability and safe operation [48], imposing less resistance to patients’ natural motion and easing their recovery. Intelligent algorithms also allow the implementation of targeted training plans [49,50], leading patients to the comprehensive recovery of sensorimotor and neuromotor function.

Finally, devices with dual control architectures, in a mirrored or leader-follower configuration [51,52], reinforce the rehabilitation process in post-stroke patients [53]. It is concluded that the implementation of intelligent algorithms could lead to the development of improved systems that prioritize individual assessment based on physiological parameters of patients, thus becoming clinical diagnostic and treatment tools that can work closely with medical specialists [54].

### 2.3. Compact, Portable, and Wearable Exoskeleton Devices

Assistive technology devices in rehabilitation should be as portable, lightweight, compact, and ergonomic as possible for users [55], allowing their inclusion in activities of daily living (ADLs) [56]. Cable-driven exoskeletons are notable due to their flexible and lightweight designs [57,58] as well as robotic exoskeletons made from additive manufacturing techniques [59,60]. Their main advantages are usability and portability, gaining versatility for training plan implementation as well as becoming key devices to support effective motor function rehabilitation.

### 2.4. Multi-Parameter Exoskeleton Devices

Electromyography (EMG), electroencephalography (EEG), electrocardiography (ECG), and other parameter measurement systems also play an important role in detecting motion intention. Some of these studies [61,62,63] report methodologies where EMG-EEG systems produce beneficial results in rehabilitation sessions with active exoskeletons. The contributions of Frolov [64] and Gordleeva [65] are relevant in the field of brain-machine interfaces, bringing an interaction where users control a robotic system even without prior training. Other approaches involve surface electromyography (sEMG) systems [56], whose signals are used to control a 1-DoF robotic exoskeleton, providing a device that improves upper limb muscle function in ADLs. When designing robust controllers with multi-parametric inputs, notable results have been achieved using EMG-based control strategies in exoskeletons for elbow rehabilitation [66].

The results of further research show that the integration of sEMG into these controllers allows for adaptive and flexible assistance [67,68], enabling active prediction of patient motion. Finally, in terms of data fusion that can improve the control of an active exoskeleton, Teleceptive Sensing is proposed as a methodology that revolutionizes the way machines acquire and process data [69].

### 2.5. AI-Based Techniques for Multimodal Pattern Detection

Some techniques based on artificial intelligent algorithms involve multi-signal processing (including EMG, EEG, ECG) for early pattern detection that can improve the development of robotic exoskeletons. Although research does not immediately reflect applications in rehabilitation, it highlights contributions related to fatigue detection [70,71,72] and recognition of complex human activity in diverse scenarios [73]. New studies have complemented traditional control techniques for robotic systems with Natural Language Processing (NLP) and voice detection techniques [74,75,76], creating exciting but early prospects for the future expansion of this topic.

## 3. Materials and Methods

**Figure 1:**
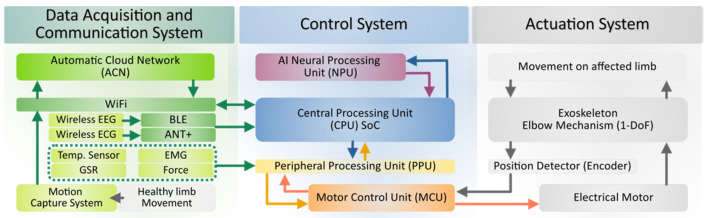
Overview diagram of the main subsystems that constitute the exoskeleton prototype.

Considering the current state-of-the-art, and based on the research objectives and a pre-established methodology [54], this section reflects the materials and methods used for the prototype development. The proposed robotic exoskeleton is aimed to support autonomous neuromotor rehabilitation processes in the arm and forearm segment (elbow joint). This development enables 1-DoF motion without requiring a fixed structure employing wearable technology. The proposed exoskeleton is composed of three main systems, as shown below in Figure 1.

The exoskeleton includes: (i) the data acquisition and communications system, which collects information from internal and external sensors, also supporting the telemetry services; (ii) the control system, which is responsible for information processing, decision making, and activation of the actuation system; and (iii) the actuation system itself, which performs the movement of the upper limb, providing feedback to the closed-loop control system.

### 3.1. Structural Design

The structural design of this prototype focuses on three main components: (i) providing a lightweight and compact portable system; (ii) allowing the design to be easily constructed or replicated; and (iii) having a modular design in which any subsystem of the exoskeleton, including its control and communication electronics, as well as the included sensors, can be attached, expanded or removed. 

**Figure 2:**
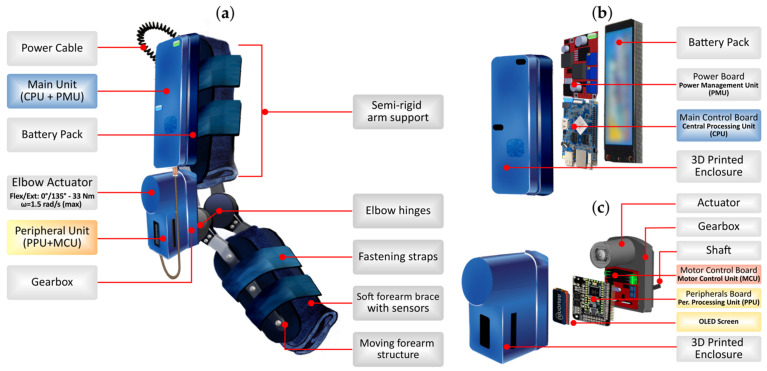
Physical design and main parts of the exoskeleton prototype: (**a**) fully assembled device (sagittal plane); (**b**) Main Unit (exploded-view drawing); and (**c**) Peripheral Unit and the actuation system (exploded-view drawing).

A summary of the physical and mechanical characteristics of the exoskeleton is presented in Table 1. Additionally, Figure 2 presented next shows a diagram containing all the functional elements that constitute the exoskeleton prototype. 

The system is mainly composed of four semi-rigid support paddles that provide lateral support to the arm and forearm segments, attached at each end to a lightweight articulated structure that allows their movement (Figure 2a: moving arm/forearm structure). An ergonomically designed wrap-around surface is fitted between the paddles, enabling the upper limb segments to be covered and attached to the main structure (Figure 2a: soft arm/forearm brace). This soft brace contains sensors (as described later), and anti-slip strips to ensure maximum grip. 

The final support on the limb is provided by fastening straps on both the arm and forearm, so the initial position of the exoskeleton remains fixed during rehabilitation or training sessions, allowing proper adjustment to multiple anatomies. Electronics and the actuation mechanism are attached to the structure using various fastening methods, including screws, rivets, or soft materials such as loop-and-hook fabric. 

The Main Unit (Figure 2b) is attached to the upper outer paddle, and consists of: (i) a 3D printed enclosure; (ii) the Power Management Unit (PMU) board, (iii) the main Central Processing Unit (CPU) board; and (iv) a battery pack that enables autonomous and wireless operation of the exoskeleton. Nearby is the Peripheral Unit and actuation system (Figure 2c) consisting of: (i) a 3D printed enclosure; (ii) the peripheral board with the Peripheral Processing Unit (PPU) and an OLED display; (iii) the Motor Control Unit (MCU) board; (iv) the electric motor; and (v) the gearbox with its final rotation shaft.

**Figure 3:**
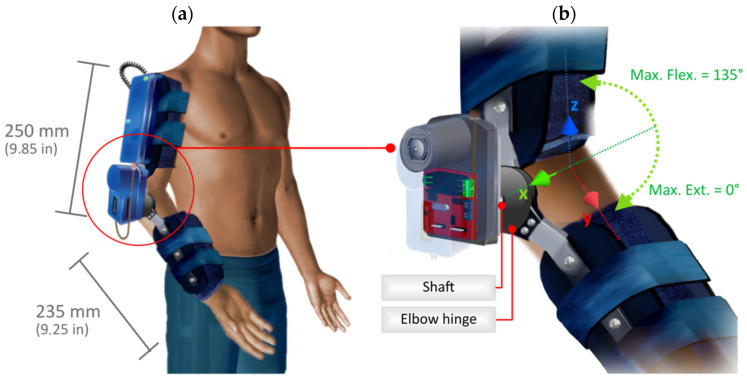
Attachment of the exoskeleton prototype to the upper limb. Part (**a**) shows the location of the exoskeleton and its size. Part (**b**) shows the actuation system in detail.

As shown in Figure 3a, this actuation system works at the elbow joint level, actively assisting the flexion and extension movement. The motion produced by the motor is transferred to the gearbox, and finally to the system structure by coupling the shaft to the elbow hinge, as shown in Figure 3b. This set-up allows the appropriate motion to be applied to the limb, providing a nominal torque of 33 Nm, with a software-defined joint amplitude range from 0° (at maximum extension) to 135° (at maximum flexion). These movements are software and physically limited at the coupling hinge for user safety.

Considering that the natural range of elbow flexion-extension motion ranges between −6° and 11° (at maximum extension) and between 130° and 154° (at maximum flexion) for healthy limbs [77], the device covers most of the amplitude range needed in rehabilitation processes. However, it is possible to extend the articular range of the exoskeleton by changing the safety and configuration parameters. The angular speed is reconfigurable, varying from 0 to 1.5 rad/s. The prototype is kept compact, with a maximum arm segment length of 250 mm and a maximum forearm segment length of 235 mm. The physical design of the exoskeleton can be considered adequate within the anatomical standards of an average adult. 

**Figure 4:**
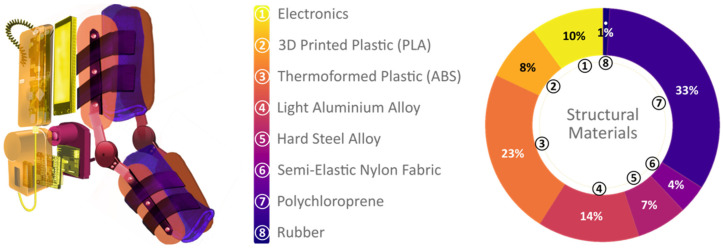
Materials used in the prototype and their percentage, proportional to the total volume.

The exoskeleton is made using different materials to generate stable support for the upper limb, yet remaining light and comfortable to wear. The prototype has a total weight of 988 g. As shown in Figure 4, the average distribution of materials per volume unit is presented.

### 3.2. Data Acquisition and Sensor Fusion

The exoskeleton prototype has a robust data acquisition and communication system, which also involves sensor information fusion techniques (see Figure 1). As part of the exoskeleton’s built-in sensors, a plurality of electronic elements are arranged throughout its structure, allowing some of its parts to be active surfaces for measuring the user’s or patient’s parameters.

On the other hand, the prototype also has the possibility of coupling different external sensors that can be wirelessly intercommunicated. These elements provide additional information about the interaction between the exoskeleton and the user, which allows further information to be collected in a rehabilitation assistance process. A summary of the included sensors is presented in Table 2.

#### 3.2.1. Peripheral Sensors

**Figure 5:**
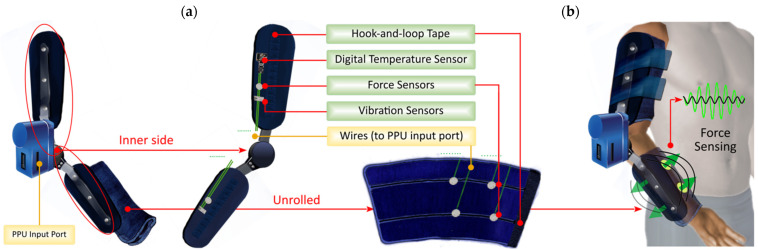
Peripheral sensors integrated into the exoskeleton. Part (**a**) shows the location of the sensors on the structure. Part (**b**) shows the force sensors that detect variations in the forearm when flexion or extension occurs.

As shown in Figure 5a, peripheral sensors are referred to as the data acquisition elements that are present in the device structure. The processing of these sensors is done by the Peripheral Processing Unit (PPU).

As shown in Figure 5a, vibration and resistive force sensors are included on the inner side of the support paddles to enrich the information about the mechanical behavior of the exoskeleton when in operation, also providing data about the pressure exerted by the exoskeleton structure when it is adjusted on the limb. A temperature sensor is also included, monitoring the temperature of the patient’s limb during training and rehabilitation activities. This is useful as it is desirable to prevent the human body from exceeding the appropriate temperature ranges during the session.

On the inner side of the soft forearm brace, a total of 4 force sensors are included: two for flexion and two for extension movements. As shown in Figure 5b, these sensors wrapped around the forearm and allow for the detection of movement intention patterns in real-time, capturing the variations through the pressure of the limbs. 

The soft arm/forearm braces are wrapped around themselves using hook-and-loop tape and are attached to the support paddles with the same fastener, making them completely removable.

Referring to the information processing, the prototype can receive and process in real-time the data coming from the sensors. For this purpose, the information (of analog or digital nature) can be provided through the input port located on the side of the PPU (Figure 5a). In addition, the PPU also provides high-priority information through an integrated OLED display (seen in Figure 2c).

#### 3.2.2. External Sensors

**Figure 6:**
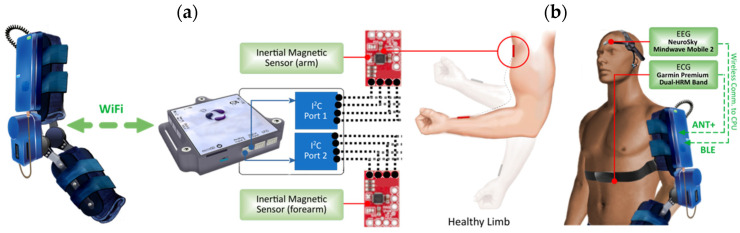
Sensors external to the robotic exoskeleton, wireless protocols. Part (**a**) shows the communication between the exoskeleton and the motion capture system. Part (**b**) shows the connection between the exoskeleton, the EEG, and ECG external sensors.

A diagram showing the different external sensors that can be attached to the exoskeleton is presented in Figure 6.

As a complement, multiple externally installed modular sensors allow the evaluation of additional user parameters. This allows a larger information volume to be fed back to the control and processing systems, extending the capabilities of the exoskeleton as a diagnostic assistance system. From this, and as will be expanded upon later, intelligent and adaptive algorithms will be able to make future decisions about mechanical behavior based on the individual user’s actions.

##### Motion Capture System

The prototype has been designed to be compatible with the motion capture system presented in [78]. This system is composed of MEMS-based inertial-magnetic sensors (IMUs), interconnected wirelessly by a separate processing unit (Figure 6a). In terms of data flow and processing, the User Datagram Protocol (UDP) is used for sending and receiving packets to the motion capture system in real-time. In general, this system is used to allow the exoskeleton to mimic (in Leader-Follower configuration) the biomechanical movement of a functional/healthy upper limb of the patient, or even from a second person (such as the physical medicine and rehabilitation professional, for example), to perform synchronous or mirror-like rehabilitation exercises.

##### ECG, EMG, and EEG Data Acquisition

The prototype is equipped with other external sensors that provide real-time information such as (i) heart rate from an electrocardiography (ECG) sensor; (ii) synchronous brain activity while executing training or rehabilitation exercises from surface electroencephalography (EEG) sensor without electrodes; (iii) muscle response over the exercising upper limb from surface electroencephalography (sEMG) sensor; and (iv) changes in electrodermal activity or sweating via a Galvanic Skin Response (GSR) sensor. Some of the equipment and interconnection with the exoskeleton is shown in Figure 6b.

Firstly, the ECG sensor allows the acquisition of heart rate wirelessly via the ANT+ protocol. The Central Processing Unit of the exoskeleton has an integrated transceiver that allows the protocol to be used. Due to its modular nature, the heart rate monitor can be removed or placed on top of a sensitive band. The incorporation of ECG allows a close recording of the cardiac behavior which the patient may experience during the use of the exoskeleton, providing event detection, such as fatigue or overexertion, generating the corresponding control actions in each case.

The EEG signal monitor allows the acquisition in real-time of different brainwaves using the Bluetooth Low Energy (BLE) protocol. The sensor consists of a convenient headband worn on the head, with an arm resting on the forehead and a connector placed on the earlobe. The raw captured signals correspond to alpha, beta, gamma, delta, and theta waves, plus 7 more signals that are related to the brain activity of each patient. The incorporation of this EEG monitoring system allows the patient’s brain activity during the use of the exoskeleton to be analyzed by intelligent processing algorithms. 

From this, mental fatigue, tiredness, or the level of neuronal activity can be detected, allowing the exoskeleton to make changes to the rehabilitation routines required to reinforce the restoration of neuromotor function.

Finally, it is possible to collect signals from an sEMG or a GSR system that can be attached to the motion capture device to extend its functionalities [78], or be directly connected to the exoskeleton through using the aforementioned protocols. The sEMG signals are useful for evidencing the patient’s muscle effort during active training sessions using the exoskeleton.

### 3.3. Control and Processing Hardware

The infrastructure that allows the control and operation of the exoskeleton are provided by specific embedded hardware elements for the required functionalities, distributed on multiple Printed Circuit Boards (PCB). A summary of the elements that compose the complete architecture is presented in Table 3.

The main embedded microcomputer system consists of a Rockchip RK3399 System-on-Chip (SoC) CPU. This unit runs a custom-compiled Linux-based Operating System (LOS), with a Preemptive Real-Time kernel. The LOS supports the execution of all the modules, whose function can vary from specific drivers for the required hardware platforms, control, processing, security, and communication algorithms. Additionally, a Neural Coprocessor (NPU) is embedded on the board and interfaced via USB. Some intelligent algorithms are also executed using the SIMD instructions of the vector coprocessor or the GPU included in the main SoC.

As for the peripheral unit, the PPU microcontroller includes an integrated digital signal processor that handles the received signal from the built-in sensors in real-time. Both the PPU and the CPU are linked via USB. The firmware of this unit is designed to collect, process, filter data from peripheral sensors and send information to the Motor Control Unit (MCU).

The main functionality of the MCU is to maintain the continuous and correct operation of the actuator, executing routines for checking the angular position of the main shaft using an encoder, as well as processing commands related to rotation, speed, torque, among others.

Finally, the system responsible for power supply and management features is the Power Management Unit (PMU) featuring an embedded power supply controller and a conversion controller. In addition, a rechargeable lithium polymer-based battery pack is also included to enable the autonomous operation of the entire prototype. 

### 3.4. Control Strategies for the Exoskeleton Prototype

This section describes the different control strategies, operation modes, and other subsystems that allow the final actuation of the exoskeleton prototype.

#### 3.4.1. Automatic Cloud Network (ACN)

This prototype is capable to send and receive telemetry, control, and processing information wirelessly over a local network or the Internet. This system is called “Automatic Cloud Network” (ACN). The advantages of ACN reside in the ability to locally or remotely manage the functionalities of the exoskeleton, such as remote control or remote parameter monitoring by a doctor or health specialist, among other technical functionalities of the prototype, allowing an external supervisor to obtain feedback from all functional subsystems. 

ACN can operate in two modes: (i) in Station (STA) mode, the device locates a known WiFi network and runs the ACN service. If no known WiFi network is found in STA mode, the exoskeleton switches to (ii) Access Point (AP) mode, where a new private WiFi network connection is created thus running the ACN service in local mode. Even if there is no Internet connection, the private connection allows for full-functionality or to set up a new WiFi station with Internet access to send and receive data remotely at a later time.

#### 3.4.2. Hierarchical Control System

**Figure 7:**
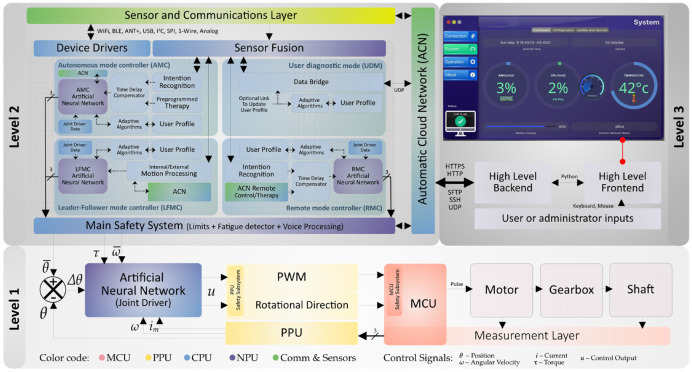
Block diagram of the exoskeleton prototype hierarchical control system.

The exoskeleton prototype is mainly operated by a hierarchical control system distributed over three levels that work together to provide the final control output. The higher the numbering, the higher the level of abstraction. Broadly defined, the hierarchical control system allows the application of routines aimed at the recovery of the motor function of the upper limb in different individuals, such as patients with neuromotor pathologies that prevent them from carrying out ADLs, or patients undergoing physiotherapy processes. As presented in Figure 7, the hierarchical control system is highly distributed, involving all elements of the data collection, information fusion, and control hardware architecture.

##### Level 1: Core Control

The exoskeleton actuation system is governed at the lowest level by the MCU, which obtains (i) feedback of the position (*θ*) of the main shaft from the onboard encoder; and (ii) current measurements (*i_m_*). This information is sent to the PPU which acts as a bridge to the CPU, where a position, velocity, and torque controller based on Artificial Neural Networks (ANN) is finally implemented, referred to as the joint driver. This driver is governed by level 2 modular controllers, producing in response a digital value consisting of the modulation percentage of a pulse-width modulated (PWM) signal and the rotational direction. The PPU decodes these control parameters, which are sent to the MCU for execution, enabling the final flexion-extension action at the elbow joint. 

The ANN-based joint driver allows three basic modes for the operation of the actuator: (i) active assistance, where the motor generates and guides the exoskeleton and user movements; (ii) active resistance, where the motor opposes the patient’s movement at percentage levels of resistance (which can be configurable from 0 to 100%); and (iii) dynamic, where the exoskeleton actively assists the limb movement, but gently rejects disturbances, for example, those arising from spastic conditions.

##### Level 2: Modular Control

This level is responsible for the control of the exoskeleton based on the system models and the strategies associated with each operation mode, allowing the generation of movement assistance and rehabilitation routines. In general, modular control combines exoskeleton dynamics with information gathered from sensor fusion and patient profile, enabling or disabling features from different intelligent controllers depending on the current operating mode.

The deployment of the different functional modules in level 2 allows the exoskeleton to operate autonomously or under guidance. In a transversal way to the different operation modes, there are intelligent algorithms that also enable the enrichment of the incoming information, such as the identification of usage patterns, predictive detection of motion intention, detection of mental and physical fatigue, multi-parametric evaluation of the user and the integration of voice commands. This modular level also establishes the different user safety and security features, which will be further elaborated on below.

##### Level 3: Control Management

The exoskeleton has a high-level management system, supported by the ACN system, that allows checking the operation of the prototype, obtaining real-time data, and configuring all the functional features of the device. The low-level communication is done through SSH, UDP, and other network protocols, while the front end uses a graphical user interface (GUI) created in Qt and Python. Among the main functionalities include the ability to configure patient profiles, monitor multimodal signals in real-time, manage pre-programmed training plans, select the operation mode and define safety parameters, as well as update the exoskeleton’s software and firmware. The management GUI is portable, running on all available operating systems.

#### 3.4.3. Operation Modes

**Figure 8:**
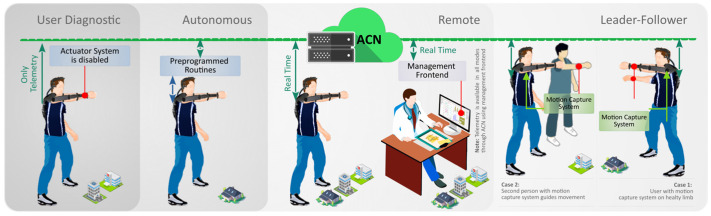
Four operation modes were designed for the exoskeleton prototype.

The different operation modes arising from the modular controllers arranged at level 2 of the control hierarchy presented above are discussed in detail. It should be noted that these modular controls act independently of each other, meaning that it is not possible to run more than one operation mode simultaneously. An illustrative diagram of the available operation modes is shown in Figure 8.

##### Autonomous Mode

In this operation mode, the exoskeleton prototype bases its movement on previously programmed rehabilitation sessions, that were stored in the device. These routines are being read locally. The control architecture of this operation mode can be detailed in Figure 7 (Level 2—“AMC” block). The objective of this mode is to execute sessions previously defined by a rehabilitation specialist or the patient itself and can be sequences of repetitive movements at specific time intervals.

In this mode, the prototype’s pattern recognition system can be used to verify the patient’s effort in the middle of the execution of a movement routine, allowing interactive sessions to be programmed where the user must be an active participant in the training. A digitally implemented time compensator is used to temporarily modify the pre-programmed sequences and allow the performance of the rehabilitation routines according to each patient’s pace (in conjunction with movement intention prediction) if necessary. Also, ACN can be used to monitor the patient’s progress, as well as to obtain telemetry of the process or save/update training plans previously performed inside the exoskeleton.

##### Remote Mode

In this operation mode, the exoskeleton bases its movement on rehabilitation sessions operated remotely and in real-time via ACN. The control architecture of this operation mode can be detailed in Figure 7 (Level 2—“RMC” block). The objective is to execute the rehabilitation actions that a specialist or physician defines for the patient or user, supporting telerehabilitation processes without losing track of the individual profile. Other features of the autonomous mode are also used in the remote mode, such as pattern recognition. In this case, the time compensator smooths the human-machine interaction retaining the ability for rehabilitation routines to be performed according to the pace of the individual patient.

Furthermore, the administration GUI is constantly updated with information from the prototype. This allows the user, the physician, and all other parties involved in the rehabilitation process to receive the same information on system behavior, user parameters, and the exoskeleton settings. The remote operation of the system, as well as the centralization of processes over the internet, allows the complete portability of the exoskeleton, where the patient can be in a hospital, rehabilitation center, or at home, while medical professionals can be anywhere.

##### Leader-Follower Mode

In this operation mode, the exoskeleton prototype will reliably perform the motion produced in the motion capture system, regardless of whether it is used by the same user or by a second person. The control architecture of this operation mode can be detailed in Figure 7 (Level 2—“LFMC” block). The objective of this mode is to achieve maximum efficiency in the recovery of the patient’s motor function by operating in a mirror-like mode. Adaptive computational algorithms ensure detailed adjustments according to the individual profile and its specific recovery progress.

In the first case, the user can control the exoskeleton using the motion capture system on a healthy limb, allowing synchronized movement in both arms. In the second case, a doctor or other person can externally guide the prototype’s movements using the motion capture system. In either situation, the control system ensures that it is operating within the parameters and settings given by the rehabilitation expert for each profile. ACN can be used to monitor patient progress, as well as to obtain telemetry, adjust system variables or work on the user’s profile.

##### User Diagnostic Mode

The objective of this operation mode is to use the exoskeleton as a user diagnostic system, collecting the patient’s multiparametric information, and optionally, creating or update the patient’s profile. In this mode, the exoskeleton will deactivate the ANN-based joint driver (Level 1 hierarchy), allowing unrestricted movement of the joint (with no active or resistive mechanical activation), while all other subsystems remain fully operational. It should be noted that in this operation mode it is not possible to send movement orders, therefore level 1 driver is limited to provide the joint position data. The feedback architecture of this operation mode can be detailed in Figure 7 (Level 2—“UDM” block). In this mode, ACN can be used to obtain telemetry, adjust system variables or user profiles, create training plans to be used in another mode of operation, perform calibration of the sensing layer, and test the prototype subsystems safely.

#### 3.4.4. Safety Systems

The exoskeleton prototype has been designed with the concept of intrinsic safety in mind: if any system or subsystem (either physical or logical) stops working or does not work properly, other components of the design can temporarily replace its functionality or disable the device for safety. Additional protective features are coded into a cross-cutting supervisory layer (referred to as “main safety system”), increasing reliability in the application of rehabilitation routines and reducing the risks associated with human factors. These aspects are detailed below.

##### Main Safety System: Cross-Cutting Supervisor

**Figure 9:**
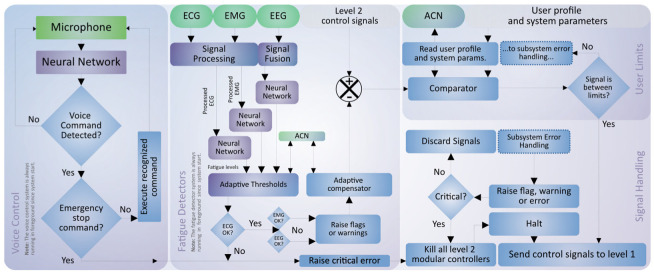
Graphical description of the logical functioning of the main safety system.

The control signals produced by the modular controllers (operation modes) are verified by the main safety system before being sent to the level 1 ANN-based joint driver. This system is a cross-cutting supervisor, independent of the control modules. Its general functionality can be seen in Figure 7, but is detailed below in Figure 9.

The main security system consists firstly of the voice control module. In this case, signals are processed by the NPU from an audio sample coming from the microphone and then analyzed in a speech recognition system based on neural networks. The model recognizes previously trained words. If a command corresponds to one of the keywords, the corresponding action is executed. The voice control module also allows an emergency shut-down with a specific command, deactivating any running controllers and halting the operation of the actuator system. This module runs in the background from the initial start-up of the exoskeleton and remains active constantly in a loop.

The second safety measure is a predictive fatigue detection system, which works with the patient’s or user’s ECG, EMG, and EEG signals. In this case, the system constantly analyses the sensor signals for signs of fatigue, which can be detected by neural networks and pre-trained adaptive comparators. In case fatigue is detected, some actions can be taken: (i) in case of cardiac or pulse anomaly, the system will completely halt as a safety measure; (ii) in case of muscular or mental fatigue (low patient attention or frequent distraction about the training session), the system can condition the signals produced by the active modular controller and change parameters such as speed, joint aperture ranges, among others.

Finally, the main safety system incorporates a comparison of the signals resulting from the previous stage against the particular limits and conditions reported in the user’s profile. This ensures that the control signals are within the individual operating parameters. If any of these stages fail, the system can decide to discard the signals, raise flags or errors, and in any critical or malfunctioning case, disable the level 1 driver from performing any action on the actuator system. In any case, all safety parameters can be configured and constantly monitored via ACN.

##### Privilege and Exception Levels

At level 2 of the hierarchical controller, the Linux-based operating system (LOS) manages access to the system’s logical and physical resources (CPU, GPU, NPU, etc.) according to a pre-defined privilege and exception level model. Some subsystems of the prototype, such as device drivers, have been coded as kernel modules. On the other hand, the algorithms that compose the different modular controls and the main security system are executed in privileged (root) mode. Finally, the ACN system and its various intercommunication components run in unprivileged user mode. In the event of a recoverable NPU or PPU failure, the CPU can warm restart the inoperative units.

##### Mechanical and Logical Safety Aspects

Physically, the prototype exoskeleton has locking pins (located in the elbow hinges) that limit the maximum flexion and extension values that can be achieved. Although their use is optional, they can provide a mechanically redundant safety system if needed.

Digitally and at level 1 in hierarchical control, the firmware encoded in the MCU and PPU has some safety features. It allows defining the maximum angle values and speed, which should match in both units. The limits are governed by the CPU but are set by whoever manages the operation of the exoskeleton, depending on the configuration of the user profile. Even if the joint driver sends out-of-bounds parameters, the PPU will reject the command. In case of PPU failure or data corruption, the MCU can also prevent motor activation if necessary. Safety settings for power management as well as battery management are managed by the PMU.

## 4. Results: Controllability and Accuracy of the Exoskeleton Prototype

This section presents the most important preliminary verification results obtained by testing the functional characteristics of the exoskeleton prototype in a controlled laboratory environment. The main objective is to gain a proof-of-concept validation of the described technologies integration, as well as a preliminary insight into the controllability and accuracy of the established hierarchical control architecture. The stability and reliability of the exoskeleton in all its aspects are correlated with patient safety and the potential feasibility of using this exoskeleton prototype in diagnosing and assisting neuromotor rehabilitation processes.

### 4.1. Wearing

**Figure 10:**
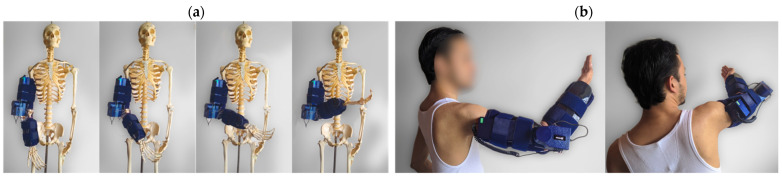
Wearing of the exoskeleton prototype. Part (**a**) shows a natural-scale skeleton for verification in the early stages of development and testing. Part (**b**) shows a human test subject wearing the prototype (for reference purposes).

The modular nature and architecture of the exoskeleton allow it to be adapted to different anatomies, making it a wearable system that is straightforward to attach or detach. Thanks to the materials used, the flexibility, and the attachment method, the exoskeleton can be put on and taken off several times without changing its mechanical properties. Figure 10 shows the placement of the exoskeleton prototype.

### 4.2. Autonomous Mode

**Figure 11:**
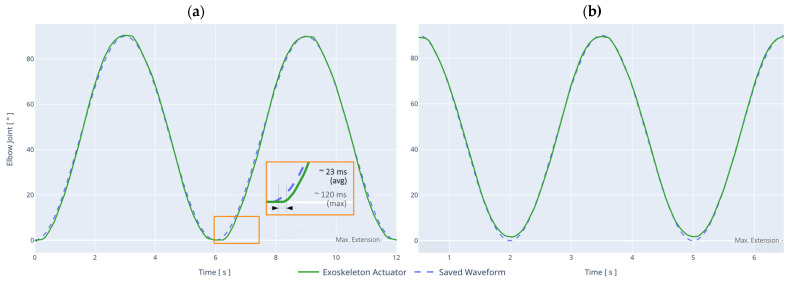
Autonomous Mode Controller (AMC) test using a sinusoidal waveform. Part (**a**) angular velocity ω = 30°/s (0.52 rad/s). Part (**b**) angular velocity ω = 60°/s (1.05 rad/s).

The test performed on the Autonomous Mode Controller (AMC) corresponds to the tracking of a sinusoidal waveform trajectory pre-programmed in the exoskeleton, where the amplitude range is between 0° (maximum extension) and 90°. The waveform is defined by a binary file, with a sampling rate of 120 Hz. The objective of this test is to determine the motion accuracy under two different speed settings, to measure the average response time of the actuation system, and to check the performance of the overall control architecture. The results are presented in Figure 11.

The results show that the test is successful, where the exoskeleton accurately tracks the programmed trajectory, preliminarily validating the hierarchical control architecture. Note that the total test duration is 30 s in each case, although Figure 11 only shows 2 cycles of the waveform.

Accurate trajectory tracking is essential for the development of complex rehabilitation routines where precise control of the patient’s or user’s joint amplitude is required. The overall root-mean-square error (RMSE) is determined to be (i) 0.3774° (6.58 × 10^−3^ rad) for ω = 30°/s (0.52 rad/s); and (ii) 1.6702° (29.15 × 10^−3^ rad) for ω = 60°/s (1.05 rad/s). 

Although the RMSE increases as a function of angular velocity, its behavior is stable over time, remaining within the same value. In the worst-case scenario, the measured RMSE was 4.3531° (75.97 × 10^−3^ rad) for ω = 86°/s (1.50 rad/s).

In terms of latency, the average response time from reading the trajectory point in the file to its execution in the exoskeleton’s actuation system is determined to be 23 ms. Due to this latency, the maximum effective update rate of the actuation system is 43 Hz on average. If the sampling frequency in the AMC is higher than this value, the ANN-based joint driver will compensate for the sample loss by maintaining waveform consistency with the lowest possible latency. In the worst-case scenario, the latency reached a maximum of 120 ms, which was compensated for in all cases.

### 4.3. Remote Mode

**Figure 12:**
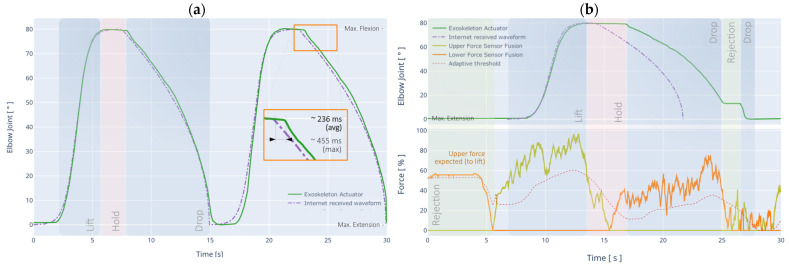
Remote Mode Controller (RMC) test using a custom trajectory sent through the Internet. Part (**a**) shows the exoskeleton response to the trajectory. Part (**b**) shows the time compensator action in the same procedure.

The objectives of the Remote Mode Controller (RMC) tests are: (i) to determine the response time of the exoskeleton and the accuracy of the movement while receiving and executing a trajectory (sequence of points) sent remotely over the Internet using the ACN system in real-time (as shown in Figure 12a); and (ii) to check the performance of the time compensator, allowing the execution of the remote rehabilitation routine according to the pace of each patient (as shown in Figure 12b).

The trajectory is designed to lift an object (active assistance), hold it for a brief moment (active resistance), and then drop the object back to its original position (dynamic). The first test of the RMC (Figure 12a) shows that the average response time of the exoskeleton is 236 ms. This time can vary depending on the quality of the Internet connection both to the exoskeleton and from the place where the trajectory is sent. In the worst case, the measured delay was around 455 ms for some of the samples, a latency that was later compensated by the controller. The overall RMSE measured was 2.5118° (43.83 × 10^−3^ rad).

In the second test (Figure 12b), the trajectory is received and stored in a temporary buffer, and then administered according to the patient’s pace (similar to the autonomous mode). In this case, the trajectory will be reconstructed when the force and EMG sensors of the exoskeleton detect the intention of the corresponding movement. RMC uses the sensor fusion information to calculate a threshold that adapts to the user’s level of interaction with the exoskeleton, recognizing that not all patients may have the same muscle strength, or it may be irregular when there are adjacent injuries or pathologies.

As can be seen in Figure 12b, since the trajectory requires lifting an object at first, the initial intention must be to flex the arm. If this does not occur, the controller produces a rejection mode. When the movement intention corresponds to the direction of the trajectory and the activation threshold is reached, the sequence is executed, where the time compensator can lengthen the sequence temporarily depending on the sensor fusion values.

A second rejection mode is reached at the end of the sequence due to the sudden change of direction in the patient’s movement intention. This is useful for controlling spasticity in the rehabilitation process. Finally, the compensator allows completion of the trajectory, and the process is resumed cyclically for the continued delivery of rehabilitation or training routines. By eliminating the dynamic action of the time compensator, the RMSE value remains practically invariant concerning the first RMC test, while the latency is approximately 32 ms (similar to that obtained in the AMC test).

### 4.4. Leader-Follower Mode

**Figure 13:**
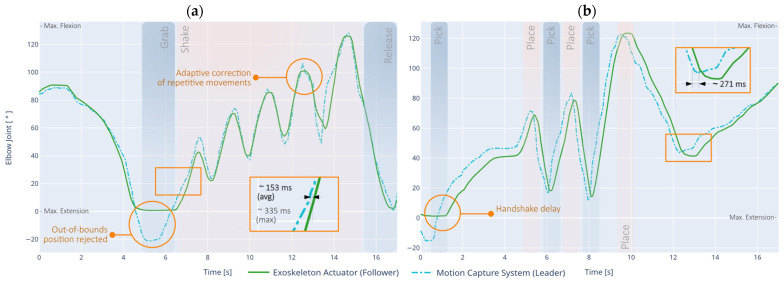
Leader-Follower Mode Controller (LFMC) test using an external motion capture system that is (**a**) connected locally in the healthy limb of the patient, and (**b**) connected via the Internet on the arm of a second person.

For this case, the Leader-Follower Mode Controller (LFMC) test corresponds to the trajectory tracking performed in real-time with the motion capture system (leader) and represented in the movement of the exoskeleton (follower). This test is used to measure the average response time of the controller, as well as the accuracy of the simultaneous movement, both in a local network connection and over the Internet. Figure 13 shows the obtained results.

In the first case (Figure 13a), the motion capture system is placed on the patient’s healthy limb and connected wirelessly via ACN in a local network, while the exoskeleton acts on the rehabilitating limb. The illustrated task consists of performing maximum arm extension to grab an object, shaking the object consistently for approximately 10 s, and returning the object to the original position.

The average response time of the exoskeleton has recorded an average of 153 ms, from the moment the motion is performed in the healthy limb until it is reflected in the exoskeleton actuator. In some cases, the latency can be as high as 335 ms, being compensated by the controller in all cases. In repetitive movements (common in ADLs), the intelligent controller detects the pattern and smooths the waveform by adjusting it consistently, which facilitates the use of the exoskeleton in everyday environments and in tasks that require increased accuracy. Because of this, the overall RMSE rises to a maximum of 9.689° (0.1691 rad), which is not necessarily a negative behavior due to the nature of the motion performed. However, this pattern detection can be disabled to achieve a more accurate match to the captured waveform, if required.

In the second case (Figure 13b), the motion capture system is placed on the limb of a second person (who may be a therapist, for example), and is wirelessly connected via ACN over the Internet. The exoskeleton acts on the rehabilitating limb of a remotely located patient. The represented task consists of pick-and-place: moving various objects from one place to another in the flexion-extension range of the patient, where all movement sequences are teleoperated.

The average response time of the exoskeleton was measured to be 271 ms on average, with the worst case being 522 ms. Since the system is connected via the internet, an increase in latency is expected. A slight additional delay is observed at the start of the session, as the parameters of the exoskeleton and the remote ACN need to be synchronized. Excluding latency, an overall RMSE of 3.429° (59.84 × 10^−3^ rad) can be obtained, which is still conducive to telerehabilitation processes.

### 4.5. Safety Concepts in Action

**Figure 14:**
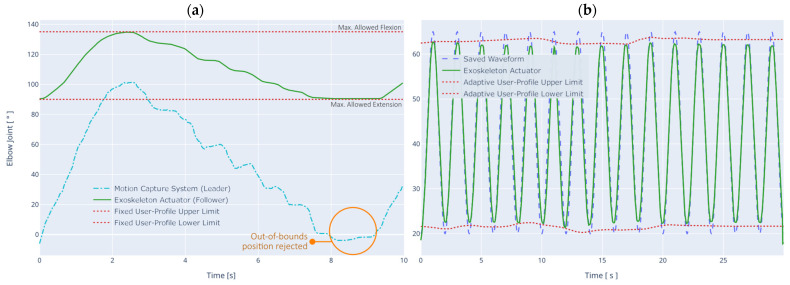
Main safety system test using (**a**) the Leader-Follower Mode with fixed user-profile limits, and (**b**) the Autonomous Mode with adaptive user-profile limits based on the fatigue detectors.

As a demonstration of the main safety system of the exoskeleton prototype, some tests have been carried out to show its operation. It should be noted that this system is always operational (unless otherwise configured) during any of the operation modes, as the control signals must be verified before continuing to the final joint driver. Figure 14 shows some of the validations performed. For the case of the first test (Figure 14a), the limits have been set in the user profile to allow for maximum flexion-extension between the ranges of 90° to 135°. After a training process, the system determines how to perform the transformation between the joint amplitude provided by the motion capture system and its correspondence between the limits set above. It is concluded that this system works correctly for all test cases. 

This function is useful in rehabilitation and training processes when the patient or user has a limited motion amplitude range, allowing precise control of the movement produced in the exoskeleton and recording the patient’s evolution as more training sessions are performed. It is also observed that when the capture system delivers outliers, the controller rejects the request and performs a movement at the limit of the allowed margins. This behavior is also observed in Figure 13a.

For the case of the second test (Figure 14b), the limits defined in the user profile can also be adaptive. With this behavior, the system can dynamically adjust the limits below the fixed limits (further restriction of movement) in case fatigue is detected by EMG, ECG, or EEG. The main safety system, through the adaptive compensator, can modify the control signals to make the final adjustments on the actuator system.

Specifically, the values set for this test are between 20° and 65°, while the controller is in Autonomous Mode, following a pre-programmed sinusoidal waveform with an angular velocity of ω = 45°/s (0.78 rad/s). It is noted that this adaptive behavior is applied in other subsystems, such as in the threshold limits for motion intention detection (shown in Figure 12b bottom).

### 4.6. Power Management and Autonomy

The total power drawn by the exoskeleton prototype is detailed in Table 4 below.

This prototype is powered by an 8000 mAh lithium-polymer battery. To maintain a more realistic estimate, the battery performance is set at 80% of the rated capacity. Taking this into account, the ideal battery life of the device is estimated to be approximately 80 min when performing activities, while it can reach up to 180 min in standby mode. Based on preliminary tests, the actual battery life of the prototype is approximately 63 min on average for active and continuous usage sessions.

## 5. Discussion

Through the application of the proposed materials and methods, a prototype exoskeleton was built that is under the desired characteristics. From the physical perspective, and compared to other previously reviewed systems such as [32,39,42,47], this prototype is more lightweight. Although there are some lighter systems [38,56,59], they are not proposed as an active elbow joint support solution. It is highlighted that the proposed prototype is fully autonomous and portable with no fixed structures for its operation compared to other systems [41,47,51,53,66,68].

On the other hand, new technologies such as cable or tendon-driven systems [36,52,57] could provide elements to improve the current proposal. The roll-up arm brace also can be improved with additional upper and lower semi-rigid supports or cuffs, as shown in [32,39,40,45], since the mechanical transfer of movement is not always effective due to the soft materials used. Although the proposed modular architecture is versatile, it still requires external assistance to position and fix the device in place due to the moving parts and the need for precise adjustment over the joint, which can be a disadvantage.

The actuator characteristics allow angular velocities above 86°/s (1.50 rad/s) which are equivalent or good compared to [39,43,47,59,66] but have been limited to maintain motion accuracy. Reducing actuator performance may reduce power consumption, but limits the execution of higher speed movements, such as presented in [32,56]. A noteworthy feature of the used actuation system is the maximum effective torque, reaching a 33 Nm peak when compared to other results [42,47,51,56,66]. The active assistance, active resistance, and dynamic operations produced by the motor and gearbox have enough power to constrain or move the patient’s limb.

An advantage of this proposal is the ability to collect and process information from a plurality of sensors, fuse such information and obtain important data for the control system. While some proposals fuse sensor data [61,62,63], most only focus on EEG [64,65] or EMG [39,67,68]. Further enhancements should be made in the wired-peripheral sensors connected to the proposed exoskeleton, as their connection is unstable due to the movement of the prototype.

The proposed tests over the intelligent control architecture establish that the average RMSE for all operating modes is approximately 3.67 ° (64,05 × 10^−3^ rad). This is considered to be a good performance when compared to [40,42,43,45,51,53] and taking into account the characteristics of the used controllers, as well as factors related to the stability of the communication link. However, the results acquired with some other intelligent control architectures [31,48,50] can provide substantial future enhancements. Also, the results shown in [32,38,46,50] are outstanding with lower average error, providing the basis for improvements in the intelligent control system. The response time is approximately 370 ms, a slow response time compared to ideally real-time systems [40,44,47].

On the other hand, the control architecture allows this proposal to be secure by having redundant mechanisms over critical processes. This security model has a unique approach among the proposals reviewed. Other contributions of this research could lie in the control modes of the device and the embedded electronics used. It is highlighted the inclusion of a dedicated AI acceleration onboard chip, which can improve NLP models compared to other developments without it [42,74,75,76]. However, in-depth studies are needed concerning the use of this chip and the detection of complex patterns such as motion prediction or fatigue detection systems [44,49,69].

Finally, the power consumption of the device is considered high due to the proposed architecture. The heat dissipation of the components could be improved. Under light loads, the CPU SoC can reach a temperature of 35 °C, while at high workloads it can be around 60 °C. In addition to a passive heatsink, an attempt has been made to cool the system with a cooling fan, but ultimately this interferes with the voice command capture microphone, which is undesirable. A comparative summary based on the characteristics assessed is presented in Table 5 below.

In general terms, and according to the previously performed tests and the experimental result observed, it can be determined that the system is sufficiently stable with and without load. This allows the prototype to be a candidate for further clinical exploration in rehabilitation processes, allowing its use in ADLs [46].

## 6. Conclusions and Future Work

This article presents an overview of the design, development, and preliminary testing of a modular and portable robotic exoskeleton that assists 1-DoF rehabilitation processes at the elbow joint. Preliminary experimental results show that the proposed architecture is optimal when tested in a controlled laboratory environment. The hierarchical control system is accurate and fast when controlling the operation of the exoskeleton in the different modes, concluding that this system meets the required stability characteristics for use as a candidate in rehabilitation and physical training applications, especially in support of ADLs.

The prototype core is made by state-of-the-art computational resources, managing the data acquisition and control functions by intelligent algorithms. The use of a dedicated AI acceleration chip and high-end embedded electronics makes this device a successful proof-of-concept, validating the technology integration. However, extensive in-laboratory evaluation on people with and without neuromotor pathologies should be carried out first and as far as possible using external measurement systems with improved accuracy. This could be done following some methodologies oriented towards qualitative and quantitative determination of therapeutic procedures [79]. It is also underlined that prior clinical validation phases should be carried out due to the need to test the prototype as an effective system to assist in upper limb rehabilitation tasks.

Aspects requiring optimization are, in order of priority: (i) decreasing the weight of the exoskeleton and improving ergonomics by using lighter materials or reducing the actuation or power supply systems; (ii) improving the audio capture method and extending the speech recognition model, including extensive testing; (iii) designing a more durable and versatile physical architecture in terms of ease of use; (iv) exploring other intelligent computational techniques that allow better utilization of sensor fusion; (v) integrating more control parameters into the high-level interface, and (vi) possibly extending the device to more degrees of freedom to allow a wider range of rehabilitation-oriented actions. 

Although future development work is extensive, the prototype could be the starting point for new intelligent diagnostic and assistive technologies for neuromotor rehabilitation, with a fully embedded processing in the exoskeleton without compromising its autonomy, ergonomics, and functionality.

## Figures and Tables

**Table 1:** Summary of the physical and mechanical characteristics of the exoskeleton prototype.

Characteristic		Value
Size	Length Arm/Forearm	Fixed: 250 mm (9.85 in)/235 mm (9.25 in)
	Width	Adjustable support paddles with semi-elastic straps to ensure arm fit
Weight	Full structure	988 g (758 g without battery pack)
Mechanics	DoF/Motion	1-DoF: Flexion–Extension at Elbow Joint
	Max. Angles	Full range actuator: 0°–300° Limited to 0°–135° for full extension—flexion
	Angular Velocity	Adjustable between 0–1.5 rad/s (0–86°/s)
	Torque	33 Nm (3.36 kg-m) at full load
Materials		Mixed, mainly polychloroprene, plastics, and light metal alloys.

**Table 2:** Summary of the internal and external sensors of the exoskeleton prototype.

Sensor Type/Quantity			Location	Protocol	Other Characteristics/Notes
Peripheral	1x	Temperature	Upper Paddle	Digital (Wired)	Maxim Integrated DS18B20, 1-Wire Bus, 12-bit
	2x	Vibration	Upper and Lower Paddles	Analog (Wired)	MEAS Spec DT Piezo Film Sensor
	2x	Force			Interlink Electronics Force Sensing Resistor 402
	4x		Soft Forearm Brace		
	1x	Encoder	MCU Board	Other (On-Board)	Used to determine the position of the motor shaft
	1x	Microphone	CPU Main Board		Used for voice commands to the exoskeleton
External	1x	Motion Capture	Healthy Arm/Other	WiFi	Motion Capture System [78], UDP Protocol
	1x	ECG	User Chest	ANT+	Garmin Premium HRM-Dual Band
	1x	EEG	User Head	BLE	NeuroSky Mindwave Mobile 2
	1x	EMG	User Forearm	Analog (Wired)	Myoware Through Motion Capture System (optional)
	1x	GSR	User Hand	Digital (Wired)	Galvanic Skin Response Sensor (optional), I^2^C Protocol

**Table 3:** Summary of the control hardware and processing architecture of the exoskeleton prototype.

Unit	Location	Tasks	Hardware Characteristics
Central Processing Unit (CPU)	Main Control PCB	Running custom Linux-based Operating System (LOS): main control, processing, safety, and communication algorithms	Rockchip RK3399 SoC: Dual-core ARM Cortex A72 + Quad-core ARM Cortex A53 + ARM Mali-T864 GPU + ARM NEON Vector Coprocessor
AI Neural Processing Unit (NPU)		Training and inference process using artificial neural networks for intelligent control structures	Gyrfalcon Technology Lightspeeur 2801S: A grid array of 168 × 168 multiply-add cores
Peripheral Processing Unit (PPU)	Peripherals PCB	Running firmware for peripheral data acquisition routines and managing the functions of the actuation system, CPU bridge to MCU	NXP Kinetis K64-120 SoC: Single-core ARM Cortex M4 @ 120 MHz
Motor Control Unit (MCU)	Motor Control PCB	Positioning of the actuation system and feedback of motion information to the CPU via the PPU	ST Microelectronics STM8S003F3 Microcontroller @ 16 MHz
Power Management Unit (PMU)	Power PCB	Provide and manage the electrical power for all exoskeleton subsystems	Rockchip RK808-D Embedded Power Supply Controller + Analog Devices LTC3780 buck-boost controller + 8000 mAh LiPo battery pack.

**Table 4:** Experimental measurements of the power draw of the exoskeleton prototype.

Unit	Power Draw without Load (mA)	Power Draw with Load (mA)
Main Control PCB	823 (5% load, all comm. modules on)	1825 (70% load, all comm. modules on)
Peripherals PCB	69	73
Motor and Motor Control PCB	1312 (idle)	2815 (avg. 50% duty cycle over time)
Power PCB and others	53	53
Total	2257	4766

**Table 5:** Summary of the results obtained in comparison with previously reviewed documents.

Criterion	This Proposal	Best Case	Worst Case	Additional Information
Weight	988 g	330 g [59]	9000 g [42]	Lower is better. The best case [59] is a wrist assist robot. There is no information on the exact weight of [38], which is an elbow assist robot.
Angular Velocity	86°/s	198°/s [32]	45°/s [59]	Higher is better. The best case [32] is a multi-DoF robot. The value specifically corresponds to the elbow actuator.
Torque	33 Nm	21 Nm [42]	3 Nm [47]	Higher is better. All systems have the appropriate torque according to the application.
Positioning Error	3.67°	0.56° [32]	5.6° [51]	Lower is better. Ref. [51] shows the RMSE associated with movement with and without a force feedback system in 3 subjects. All results are average errors.
Response Time	370 ms (avg.)	-		Although there are different systems designed with real-time applications, direct comparisons are not possible due to differences in the evaluation approach.

## Data Availability

Not applicable.
